# Chronic Subdural Hematoma in a Patient With Long‐Standing Syphilis

**DOI:** 10.1002/ccr3.72611

**Published:** 2026-04-24

**Authors:** Jose Marino, Vratko Himic, Felipe Monteiro, Joacir G. Cordeiro

**Affiliations:** ^1^ Department of Neurological Surgery University of Miami Leonard M. Miller School of Medicine Miami Florida USA

**Keywords:** chronic subdural hematoma, intracranial hemorrhage, middle meningeal artery embolization, neurosyphilis, syphilis

## Abstract

Chronic subdural hematoma can rarely occur in younger adults without trauma or coagulopathy. Comprehensive imaging to exclude vascular malformations, combined with burr hole evacuation and middle meningeal artery embolization, can achieve excellent outcomes. Long‐standing systemic infections may theoretically contribute to vascular fragility, but causality remains speculative.

## 1 Introduction

Syphilis is a sexually transmitted infection caused by the spirochete *Treponema pallidum* , characterized by a complex clinical course that includes primary, secondary, and tertiary stages, affecting multiple organ systems and often referred to as the “great imitator” due to its diverse manifestations [1, 2]. In late stages, untreated infection may lead to chronic inflammatory changes in the cardiovascular and nervous systems, including endarteritis and small‐vessel involvement, which can compromise vascular integrity and predispose to cerebrovascular complications such as ischemic stroke and, less commonly, intracranial hemorrhage [3, 4].

Chronic subdural hematoma (CSDH) is a type of brain hemorrhage characterized by the accumulation of blood products between the dura mater and arachnoid membrane that typically persist for over 3 weeks. It predominantly affects the elderly, often following mild to moderate cranial trauma, although it can also arise from other factors such as coagulation disorders or the use of anticoagulants [5, 6, 7].

While no direct exclusive link has been described between these two conditions, it is feasible that the vascular changes induced by long‐standing infection could potentially make a patient more vulnerable to hemorrhagic events such as chronic subdural hematomas. Chronic subdural hematomas have never been directly related to syphilis, with only one case suggesting this association [8].

## 2 Case History

A 47‐year‐old female with a history of hypertension and a nine‐year history of syphilis presented to the emergency department with a gradual‐onset, progressive headache that began while walking her children to school 2 days prior. The headache was associated with nausea, pain radiating down her neck, persistent vomiting, and dizziness. She denied any history of trauma, including being hit, punched, or any recent falls, and reported no other significant medical conditions.

On initial examination, she was alert, fully oriented to person, place, time, and situation, and in no acute distress. Vital signs were within normal limits except for elevated blood pressure of 157/89 mmHg. Neurological examination revealed intact coordination, motor function, speech, and gait. Glasgow Coma Scale score was 15.

**FIGURE 1:**
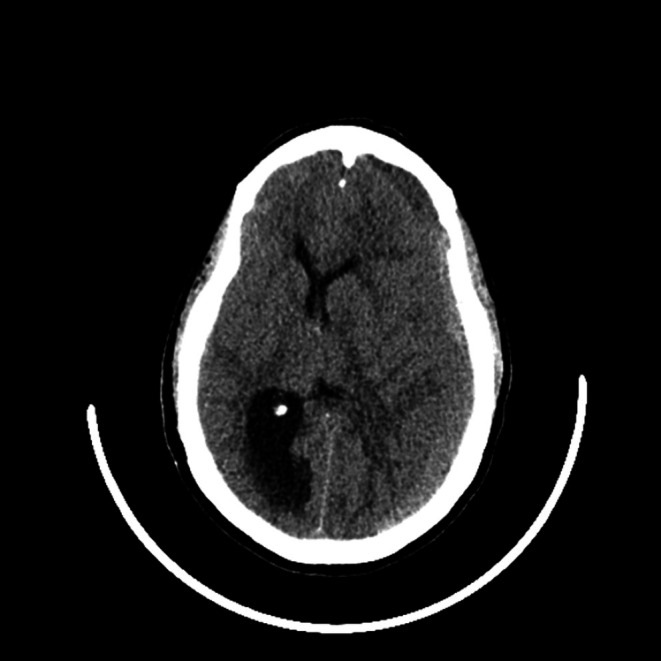
Pre‐operative CT axial scan demonstrating a large, holohemispheric, mixed density extra‐axial fluid collection with associated significant mass effect, midline shift and left‐sided uncal herniation.

Laboratory evaluation was largely unremarkable, including normal coagulation studies, although syphilis serology remained positive with reactive IgG/IgM and an RPR titer of 1:64. On arrival, non‐contrast CT of the brain demonstrated a large left holohemispheric mixed‐density extra‐axial collection consistent with acute‐on‐chronic subdural hematoma, with significant mass effect, midline shift, and early left uncal herniation (Figure 1). No abnormal enhancement was observed on contrast‐enhanced imaging. Initially, neurosyphilis was considered, and Infectious Disease recommended cerebrospinal fluid analysis; however, lumbar puncture was contraindicated due to the herniation risk.

## 3 Differential Diagnosis, Investigations and Treatment

Advanced imaging performed the following day included CT angiography of the head and neck, MRI of the brain, and CT of the cervical spine. CTA demonstrated patent anterior and posterior circulation arteries without aneurysm, arteriovenous malformation, or dural fistula. Mild tortuosity and ectasia of the high cervical internal carotid arteries were attributed to underlying hypertension rather than vasculopathy. MRI revealed no abnormal parenchymal or leptomeningeal enhancement, and cervical spine imaging demonstrated only mild spondylotic changes, effectively excluding structural vascular abnormalities as a source of hemorrhage.

The patient underwent left‐sided burr hole craniotomy for evacuation of the subdural hematoma, with thorough irrigation of the subdural space and placement of a subgaleal Jackson‐Pratt drain. Intraoperative subdural fluid was collected for microbiologic analysis, including bacterial, fungal, and acid‐fast bacilli cultures; all returned negative. Cerebrospinal fluid analysis revealed no evidence of neurosyphilis, effectively ruling out an infectious etiology. Empiric intravenous Penicillin G at 24 million units per day via continuous infusion was administered for 14 days, resulting in a decrease of RPR titer from 1:64 at admission to 1:32.

## 4 Conclusion and Results

**FIGURE 2:**
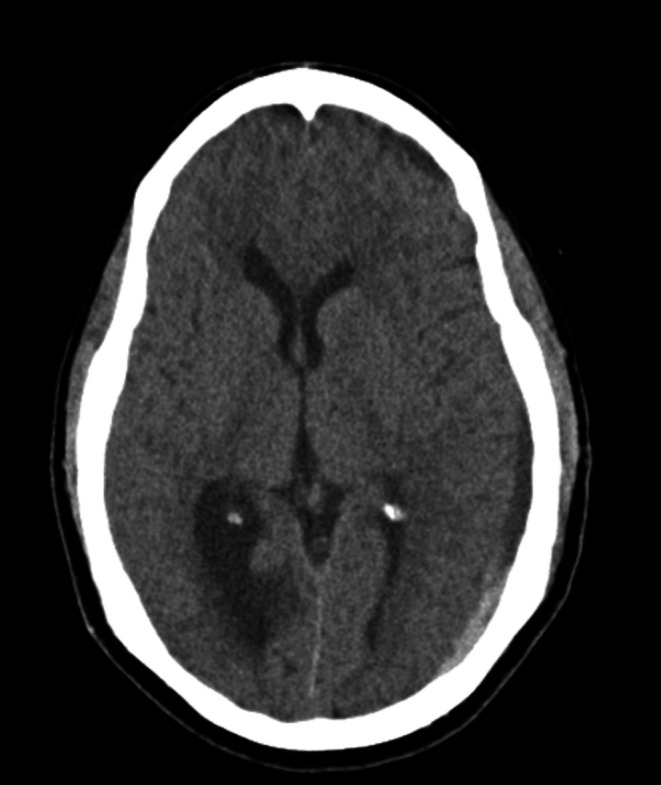
Post‐operative CT axial scan at post‐operative Day 1 showing interval decrease in size of the left subdural hematoma with associated decrease in mass effect and subfalcine herniation. Stable postsurgical changes following a burr hole for subdural evacuation.

**FIGURE 3:**
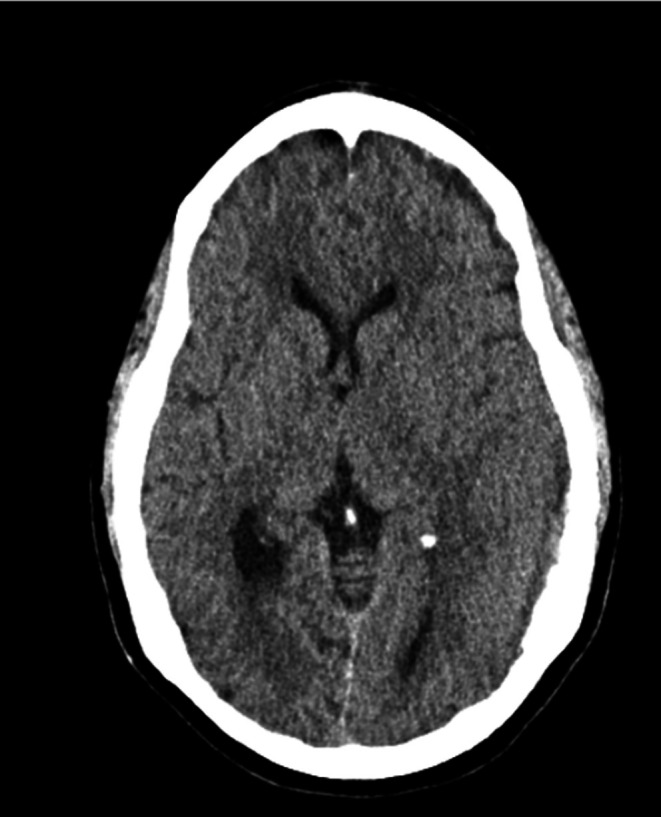
Post‐operative CT scan showing no significant change in the mostly chronic left and mostly posterior convexity subdural hematoma measuring up to 11 mm on re‐admission following fall at POD 18.

Postoperatively, CT on POD 1 demonstrated reduced mass effect and satisfactory hematoma evacuation (Figure 2). On POD 14, middle meningeal artery embolization was performed, and the patient was discharged home on POD 17. On POD 18, while at home, she experienced a seizure and a ground‐level fall, prompting return to the emergency department. CT imaging was unremarkable (Figure 3), and she was initiated on levetiracetam 750 mg twice daily. Over the following days, her neurological status returned to baseline. At outpatient follow‐up on POD 30, the patient remained seizure‐free and asymptomatic.

This case illustrates a chronic subdural hematoma in a relatively young adult without trauma or coagulopathy, in the context of long‐standing syphilis. Advanced imaging excluded vascular malformations or aneurysms, and surgical management with burr holes and middle meningeal artery embolization was effective. The presentation highlights the potential, though unproven, contribution of chronic vascular changes associated with longstanding infection to intracranial hemorrhage while effectively ruling out neurosyphilis and provides a framework for discussion of differential diagnosis, vascular fragility, and evidence‐based management strategies for atypical chronic subdural hematoma.

## 5 Discussion

Chronic subdural hematoma (CSDH) is a prevalent neurosurgical condition, with reported incidence rates ranging from 1.72 to 20.6 per 100,000 individuals annually, and particularly high rates of up to 58 per 100,000 in the elderly population [9, 10]. The condition often arises from head trauma, anticoagulant use, and advanced age, with a mean patient age of approximately 65 years [11]. Spontaneous CSDH in younger adults is rare, and when it occurs, comorbidities such as hypertension, vascular abnormalities, or previous CNS infections may contribute to pathogenesis [12, 13, 14].

In our patient, longstanding syphilis raised the theoretical possibility of neurosyphilis contributing to vascular fragility; however, lumbar puncture was contraindicated initially due to the presence of uncal herniation, and subsequent CSF analysis ruled out active neurosyphilis. Neurosyphilis can manifest with meningitis‐like symptoms including headache, neck pain, and nausea, and may cause vascular complications such as arteritis, infarction, or, rarely, hemorrhage [15, 16]. Our patient experienced headache, nausea, and dizziness, but no meningeal signs were present, and advanced imaging did not reveal vascular abnormalities, supporting a non‐infectious etiology for her CSDH.

Surgical management with burr hole craniotomy remains the standard of care for symptomatic CSDH, aiming for hematoma evacuation and reduction of mass effect [5, 6]. Nevertheless, recurrence rates can be significant, particularly in patients with atypical risk factors, necessitating adjunctive therapies. In our patient, postoperative rebleeding prompted middle meningeal artery embolization (MMAE), a procedure increasingly supported by recent randomized controlled trials and guidelines as an effective adjunct to reduce recurrence risk [9, 17, 18].

MMAE targets the vascular supply to the dura, reducing the source of microhemorrhages that contribute to hematoma recurrence. Multiple studies, including systematic reviews and RCTs, demonstrate that MMAE decreases the need for repeat surgical interventions and is safe even in patients with atypical bleeding risk factors, making it a reasonable strategy in our patient [19, 20, 21]. The procedure was successfully performed on POD 14, after which the patient remained seizure‐free and asymptomatic at follow‐up.

This case underscores that in younger adults presenting with CSDH without trauma or coagulopathy, consideration of underlying vascular fragility, including chronic inflammatory or infectious causes may guide both diagnostic workup and surgical planning. While longstanding syphilis may theoretically predispose to vascular changes, our findings emphasize that careful imaging and hematologic evaluation are essential to rule out active neurosyphilis or other structural causes. Moreover, MMAE offers a valuable adjunctive intervention to prevent recurrence, particularly in patients with atypical risk profiles, and its use is increasingly supported by high‐level evidence [18].

In summary, our patient's presentation demonstrates: (1) the occurrence of CSDH in a relatively young adult without trauma; (2) the importance of ruling out neurosyphilis and other structural vascular abnormalities; and (3) the utility of MMAE in preventing recurrence in cases with atypical risk factors. This approach aligns with contemporary evidence‐based management and supports the expanding role of endovascular adjuncts in complex CSDH cases.

Chronic subdural hematoma can occur in younger adults without trauma or coagulopathy. Thorough imaging to exclude vascular malformations, careful consideration of underlying systemic conditions, and combined management with burr hole evacuation and middle meningeal artery embolization can achieve excellent outcomes. Long standing systemic infections may contribute to vascular fragility, but causality remains speculative.

## Author Contributions

**Jose Marino:** investigation, writing – original draft, writing – review and editing. **Vratko Himic:** investigation, methodology, validation, visualization, writing – review and editing. **Felipe Monteiro:** investigation, methodology, project administration, supervision, validation, writing – original draft, writing – review and editing. **Joacir G. Cordeiro:** project administration, supervision.

## Funding

The authors have nothing to report.

## Ethics Statement

All procedures performed in this study were conducted in accordance with institutional and international ethical standards.

## Consent

Written informed consent was obtained from the patient for publication of this case report and any accompanying images.

## Conflicts of Interest

The authors declare no conflicts of interest.

## Data Availability

Data available on request from the authors.
